# Nitric Oxide-Related Oxidative Stress and Redox Status in Health and Disease

**DOI:** 10.1155/2014/129651

**Published:** 2014-07-23

**Authors:** Darko Modun, Daniela Giustarini, Dimitrios Tsikas

**Affiliations:** ^1^Department of Basic and Clinical Pharmacology, University of Split School of Medicine, 21000 Split, Croatia; ^2^Department of Life Sciences, University of Siena, 53100 Siena, Italy; ^3^Institute of Clinical Pharmacology, Hannover Medical School, 30625 Hannover, Germany

The molecular oxygen or dioxygen (O_2_) molecule has 6 molecular orbitals (MO) in its triplet state, of which the two *π*_2p_* MO contain each one electron. Thus, O_2_ has two unpaired electrons (indicated by a dot ^•^); that is, O_2_ is an uncharged diradical molecule: ^•^O–O^•^. An electron that is donated by another molecule is incorporated in one of the two *π*_2p_* MO of the O_2_ molecule; that is, one *π*_2p_* MO contains a single unpaired electron. Thus, 1-electron reduction of O_2_ yields a negatively charged radical species, that is, the superoxide radical anion (O_2_^−•^). Subsequent 1-electron reduction of O_2_^−•^ yields a doubly negatively charged species, that is, the peroxide anion (O_2_^2−^) which possesses a nonoccupied *σ*_2p_* MO. Intake of two electrons by the peroxide anion does not form a stable molecule but the bond between the two O atoms breaks to yield two O^2−^ ions which are protonated to form water.

Nitric oxide (NO) is an important signaling molecule with multiple pivotal roles in the cardiovascular and neural systems, as well as in inflammatory response. Due to its unpaired electron, NO is a free uncharged radical, with the unpaired electron being closer to the nitrogen atom of the NO molecule: N^•^=O. The high affinity of N^•^=O and ^•^O–O^•^ to many heme groups-containing proteins, notably hemoglobin, and enzymes, predominantly soluble guanylyl cyclase (sGC), determines both the metabolic fate and the biological activity of NO. Nitric oxide tremendously activates the sGC which catalyses the formation of cyclic guanosine monophosphate (cGMP) from guanosine triphosphate (GTP). N^•^=O's high affinity to oxyhemoglobin (HbFeO_2_) and its rapid Fe-catalysed oxidation to nitrate (NO_3_^−^) make direct measurement of NO almost impossible in living organisms (see reaction *(R1)*) [1]. In heme groups-free aqueous media, authentic NO may exist for several minutes, with nitrite (NO_2_^−^) being its major autoxidation product (see reaction *(R2)*) [1]. Under certain conditions, circulating and excretory nitrite and nitrate are useful indicators and measures of NO synthesis in humans [2]. Many different storage forms of NO have been suggested, with the most important being nitrite and* S*-nitrosothiols (RSNO). In addition to possessing vasodilating and antiplatelet activity,* S*-nitrosothiols can modify protein thiols by* S*-nitrosation and/or* S*-thiolation, thereby altering their inherent activity [3]:

(R1) $$\begin{array}{c} {\text{HbFe}}^{\left(\text{II}\right)}O_{2}+\text{N}O⟶{\text{HbFe}}^{\left(\text{III}\right)}+{{O\text{N}O}_{2}}^{-} \end{array}$$

(R2) $$\begin{array}{c} 4\text{N}O+O_{2}+2{\text{H}}_{2}O⟶2{O\text{NO}}^{-}+2{O\text{N}O}^{-}+4{\text{H}}^{+} \end{array}$$

Oxidative stress, that is, the imbalance between production of reactive oxygen species (ROS) and/or reactive nitrogen species (RNS) on the one side and antioxidative defense on the other side, is related with numerous diseases, along with some specific conditions such as hyperoxia, ageing, and physical exercise [2]. The direct damaging result of oxidative stress is oxidation of lipids, proteins, and DNA, all of which endanger cell homeostasis. ROS are formed from incomplete reduction of O_2_. As mentioned above, intake of one electron (e^−^) by one O_2_ molecule yields the ROS superoxide radical anion O_2_^−•^ (see reaction *(R3)*). The superoxide radical anion itself strongly tends to receive another electron to form the much more stable peroxide dianion (see reaction *(R4)*) which is protonated to form hydrogen peroxide (HO–OH, H_2_O_2_), another ROS.

When O_2_^−•^ and N^•^=O meet each other, the unpaired electron of the occupied *π*_2p_* MO of N^•^=O is transferred to the singly occupied *π*_2p_* MO of O_2_^−•^; that is, the N atom is oxidized from the oxidation state +2 to the oxidation state +3, while the O atoms of O_2_^−•^ are reduced (from the oxidation state −1/2 to −1). This extremely rapid reaction (see reaction *(R5)*) yields the RNS peroxynitrite (O=N–O–O^−^). Because the two O atoms of the peroxide group of peroxynitrite are incompletely reduced and the N atom of peroxynitrite is incompletely oxidized, peroxynitrite is highly reactive at physiological pH values. Intramolecular transfer of two electrons from the N atom to the peroxide group and its rearrangement yields nitrate (see reaction *(R6)*). In the presence of other biomolecules such as reduced glutathione (GSH; see reaction *(R7)*) or tyrosine (TyrH; see reaction *(R8)*) the two missing electrons are provided by the biomolecules which themselves are oxidized, for instance, GSH to glutathione disulfide (GSSG) and TyrH to 3-nitro-tyrosine:

(R3) O•–O•+e−⟷O•–O−

(R4) O•–O−+e−⟷O−–O−

(R5) O−–O•+N•=O⟷O−–O–N=O

(R6) O−–O–N=O⟶O(O)NO−

(R7) 2  O−–O–N=O+2GSH ⟶2  O−–N=O+GSSG+H2O2

(R8) O−–O–N=O+TyrH⟶O–(O=)N–Tyr+HO−

Modifications of biomolecules induced by ROS and/or RNS may alter the physiological function of the biomolecules and may have severe consequences for the organism [4]. In addition, oxidative stress may also reduce nitric oxide's bioavailability, because oxidative reaction products of NO including nitrite and peroxynitrite are only very ineffective sources for NO. Furthermore, oxidative stress may cause uncoupling of the endothelial nitric oxide synthase (eNOS), thus diminishing NO synthesis/bioavailability and aggravating NO-dependent oxidative stress. Excessive, uncontrolled, and unmanaged oxidative stress is certainly hazardous to humans and leads to diseases. Therefore, the prooxidative and antioxidative state in cells and tissues determines decisively synthesis and bioactivity not only of NO, but also of* S*-nitrosothiols and nitrite in health and disease, as well as in different conditions such as physical exercise and smoking.

In consideration of the eminent importance of NO-related oxidative stress and redox status in health and disease, we organized the present special issue. We are very pleased to present to the readership of the journal and to the general scientific community interested in this topic this special issue.

This thematic volume includes review and research articles. In their review paper, M. A. Abdelmegeed and B.-J. Song describe approaches for identifying nitrated proteins and studying their roles in promoting liver diseases and discuss translational research applications. S. Savvanis et al. report that sildenafil (Viagra), an inhibitor of the phosphodiesterase (PDE) isoform 5, has beneficial effects in a rat model of liver ischemia/reperfusion which is associated with elevated oxidative stress. Obstructive sleep apnea (OSA) is considered an independent risk factor for cerebrovascular and cardiovascular diseases. Paradoxically, hypoxia, which is a major pathophysiological feature of OSA, can enhance oxidative stress. In their review article, M. Badran et al. discuss the role of oxidative stress in OSA and its causal effect on endothelial dysfunction by interaction with NO in animal models and in humans. In patients with microvascular angina, B. Porro et al. investigated the interrelationship of NO and oxidative stress. Their study indicates that altered microvascular bed is associated with impaired capacity of red blood cells to produce NO, presumably due to elevated oxidative stress on the basis of the erythrocytic GSSG/GSH molar ratio as measured by LC-MS/MS.

With respect to the L-arginine/nitric oxide, children are not small adults [5]. N. K. Kanzelmeyer et al. quantified the L-arginine/nitric oxide in children with haemolytic-uraemic syndrome (HUS) and healthy controls by GC-MS. An interesting finding of this study was the close positive correlation between plasma nitrate and plasma free haemoglobin concentration. The authors discuss that elevated free haemoglobin in plasma of HUS children can not only oxidize NO to nitrate outside of the erythrocytes, but also increase oxidative stress. K. Pimková et al. quantified circulating aminothiols, nitrite, nitrate, and malondialdehyde, a widely used biomarker of oxidative stress, in patients suffering from myelodysplastic syndromes (MDS) in the context of clinical outcomes and as a consequence of iron overload. The authors found that oxidative stress is elevated in MDS, presumably independent of iron overload. These findings may bring new insight into the problematic nature both of MDS and oxidative stress.

C. M. O. Volpe et al. found that the production of NO, IL-6, and TNF*α* in cultured palmitate-stimulated PBMNCs or in plasma from type 2 diabetes mellitus patients or nondiabetic controls is elevated due to hyperglycaemia which is generally associated with elevated oxidative stress. R. Carnevale et al. explored an ex vivo experimental model in humans, in which blood cells are activated to produce ROS via NOX2, namely, the catalytic subunit of NADPH oxidase. The Steen solution, a physiological human serum albumin- and dextran-containing salt solution that is especially used in lung transplantation, was found to possess antioxidant properties via downregulation of NADPH oxidase activity and to enhance NO production.* N*-[3-(Aminomethyl)benzyl]acetamidine (1400 W) is considered a highly selective inhibitor of inducible nitric oxide synthase (iNOS). A. Mertas et al. investigated the effect of 1400 W on NO, IL-12, and TNF*α* production by LPS and TNF*γ* activated J774A.1 macrophages. The authors concluded that the potency and selectivity of 1400 W as an inhibitor of iNOS and cytokine release modifier are encouraging for therapeutic use of 1400 W.

Paracetamol (acetaminophen) is a widely used analgesic and antipyretic drug. Paracetamol readily reacts with RNS including peroxynitrite to form 3-nitro-paracetamol and di-paracetamol, in analogy to tyrosine. D. Tsikas et al. demonstrated by LC-MS/MS and GC-MS/MS the appearance of 3-nitro-paracetamol and di-paracetamol in plasma and urine samples of healthy subjects who received orally a single 500 mg paracetamol tablet and suggested a novel human model of oxidative stress based on oral paracetamol administration [6]. Application of such a model of oxidative stress to humans requires that paracetamol does not alter oxidative stress. Therefore, this group investigated the effects of paracetamol in vitro and in vivo studies in humans on the activity of enzymes that are known to produce superoxide. A. Trettin et al. found that paracetamol does not change oxidative stress, even not at the suprapharmacological single oral dose of 3 g in healthy male subjects. The utility of the paracetamol model to measure oxidative stress in health and disease remains to be demonstrated.

In summary, the present special issue assembles information from studies performed in cell systems in vitro, in animals and in human studies. The articles collected in the special issue address current standing and progress in the area of oxidative stress in relation to NO, its implication in disease, and the effect of antioxidants and drugs on NO-related dysfunction. Included articles also address models and mechanisms of NO-related oxidative stress in vitro, in animals and in humans, and provide new ideas and concepts to better understand the complex and challenging nature of oxidative stress. Delineating mechanisms are essential to effectively prevent oxidative stress and to specifically improve intra- and extracellular redox status, that is, where really required, without affecting pathways nonrelated to NO.

Before closing the editorial, we would like to express our sincere thanks to the authors for their valuable contributions and to the reviewers who helped a lot to improve the quality of the articles published herein. We hope that this work will be of help in the exciting and challenging area of NO-related oxidative stress.

*Darko Modun* *Darko Modun* *Daniela Giustarini* *Daniela Giustarini* *Dimitrios Tsikas* *Dimitrios Tsikas*
